# Patient experience in community pharmacy services: A scoping review

**DOI:** 10.1002/jgf2.70040

**Published:** 2025-06-24

**Authors:** Chiho Kaneko, Shota Suzuki, Ibuki Tanaka, Haruna Hiruma, Hiroshi Okada

**Affiliations:** ^1^ Department of Social & Community Pharmacy, School of Pharmaceutical Sciences Wakayama Medical University Wakayama Japan

**Keywords:** community pharmacy, patient experience, person‐centered care, scoping review

## Abstract

**Background:**

The role of pharmacists in community settings is expanding, and understanding patient experience– an essential indicator of person‐centered care–is crucial for improving pharmacy service quality. However, the extent to which patient experience has been studied in community pharmacies remains unclear. This scoping review aimed to explore the scope of research on patient experience in community pharmacies.

**Methods:**

This review followed the Preferred Reporting Items for Systematic Reviews and Meta‐Analyses extension for Scoping Reviews guidelines. A comprehensive literature search was conducted in October 2024 using PubMed, Web of Science, and Google Scholar with predefined keywords, including “patient‐centered,” “experience,” and “community pharmacy.”

**Results:**

Of the 121 records identified, 15 studies, published between 2008 and 2024, met the eligibility criteria. These studies were conducted in Australia (*n* = 4), the United States (*n* = 3), and the United Kingdom (*n* = 3), with six studies published in non‐English languages. Study methodologies included qualitative (*n* = 7), quantitative (*n* = 5), and mixed (*n* = 2). Four studies used patient‐reported experience measures (PREMs). Terminology for study participants varied, with “patients” (*n* = 10), “consumers” (*n* = 3), and “customers” (*n* = 1). In addition, one study included pharmacists (*n* = 1) and another included caregivers (*n* = 1).

**Conclusion:**

This review emphasizes advancing person‐centered care in pharmacy practice, with a growing focus on patient experience in community pharmacies. Future studies should develop and implement patient‐reported experience measures tailored to different social contexts to enhance care and service evaluation.

## 1 INTRODUCTION

The role of pharmacists is expanding globally, extending beyond traditional dispensing services to encompass a wide range of responsibilities, including chronic disease management, vaccination administration, self‐care promotion, and disease prevention.^1^, ^2^ Concurrently, there has been a major shift toward interpersonal services and person‐centered care.^3^ In this context, enhancing the quality of care and services provided by community pharmacies has become a critical challenge. To foster an environment that encourages active patient participation in their own care, it is essential to accurately identify their preferences and needs, and designing healthcare services accordingly.^4^ Active patient engagement in healthcare influences both the acceptability and sustainability of treatment; thus, a thorough understanding of patient preferences, expectations, and experiences is crucial.^5^

Traditionally, “patient satisfaction” has been the primary outcome measure for evaluating healthcare services, including those provided by community pharmacies. Scales designed to assess the perspectives of patients on the effectiveness of care are known as “patient‐reported outcome measures (PROMs).^6^” To date, PROM‐based patient satisfaction surveys have been conducted across diverse patient populations, ranging from general pharmacy customers to individuals with specific medical conditions.^7^ However, PROMs have not been widely applied to pharmacy services, and measurement tools are often developed independently for individual studies, raising concerns about their validity and reliability. In addition, patient satisfaction surveys can be influenced by factors such as provider behavior and patient expectations regarding treatment outcomes.^6^, ^8^

Recently, the concept of “patient experience” has emerged as a more comprehensive alternative to patient satisfaction.^5^ Unlike patient satisfaction, which reflects overall impressions, patient experience focuses on specific interactions encountered throughout the care process.^9^ Measurement tools that allow patients to objectively report their experiences and assess healthcare service quality from a person‐centered perspective are known as “patient‐reported experience measures (PREMs).^6^” Outside the field of pharmacy, studies on patient experience have grown annually, covering diverse areas, such as emergency medicine, nursing, and patient safety.^10^, ^11^, ^12^, ^13^ However, the extent to which patient experience has been studied and PREMs have been implemented in community pharmacy settings remains unclear.

This study aimed to conduct a scoping review to explore the current state of research on patient experience in community pharmacies. Specifically, it sought to (1) determine the extent of research on patient experience in community pharmacy settings and (2) assess the integration of PREMs into community pharmacy practice.

## 2 METHODS AND MATERIALS

### 2.1 Study design and protocol

This scoping review followed the Preferred Reporting Items for Systematic Reviews and Meta‐Analyses extension for Scoping Reviews guidelines.^14^ Its protocol was developed using a template provided by the Joanna Briggs Institute.^15^ The PCC framework (Population, Concept, and Context) was applied to define eligibility criteria as follows:

- Population—pharmacy patients.
- Concept—patient experience associated with community pharmacy service; review, prescribe, dispense, and administer.^16^
- Context—encompassing services provided within community pharmacies.

### 2.2 Eligibility criteria

The research team conducted multiple discussions to establish and finalize the inclusion and exclusion criteria. No restrictions were imposed on publication year.

#### 2.2.1 Inclusion criteria

Studies were included if they examined patient experience and focused on community pharmacies or community pharmacists.

#### 2.2.2 Exclusion criteria

Studies were excluded if they did not explore the patient experience of pharmacy users or if they primarily focused on patient experience. In addition, studies that were unavailable in a full‐text format or were published in languages other than English or Japanese were excluded.

### 2.3 Information sources and search

In October 2024, the researcher (CK) conducted a comprehensive literature search using PubMed, Web of Science, and Google Scholar. The search included the following terms: patient‐centered/client‐centered/consumer‐centered/customer‐centered or patient‐reported/client‐reported/consumer‐reported/customer‐reported, experience, community pharmacy or community pharmacist, service/program/intervention/counseling/consultation, and investigation/survey/research/assessment/evaluation/measurement/trial/study/analysis. These terms were searched as MeSH terms and/or keywords, depending on the database.

To improve the accuracy and comprehensiveness of the search strategy, researchers with expertise in pharmacy practice (SS and HO) reviewed and refined the keywords and search structure. The detailed search strategy is presented in Appendix Table A1.

### 2.4 Selection of sources of evidence

The retrieved records were imported into Rayyan^17^ and duplicates were removed before the primary screening. During the primary screening, records from PubMed and Web of Science were reviewed based on titles and abstracts, whereas those from Google Scholar were screened based on titles and document types to determine eligibility. Subsequently, a secondary screening was conducted on the full texts of the studies that passed the primary screening.

These screenings were performed independently by three researchers (CK, IT, and HH). In cases of discrepancies, final decisions were made through discussions with additional researchers (SS and HO).

### 2.5 Data charting process and data items

For each included study, key bibliographic information was extracted, including the title, authors, publication year, country, language, research methodology, and key findings. For studies focusing on PREMs, additional details were extracted, such as the scope of measurement and the number of items in the scale.

The extracted data were managed in Microsoft Excel. Input provided by researchers was reviewed and discussed by the broader research team until a consensus was reached.

### 2.6 Synthesis of results

Consistent with the objectives of this review, studies were categorized based on study methodology and patient experience. Key issues for future studies were also examined.

## 3 RESULTS

### 3.1 Selection of sources of evidence

**FIGURE 1:**
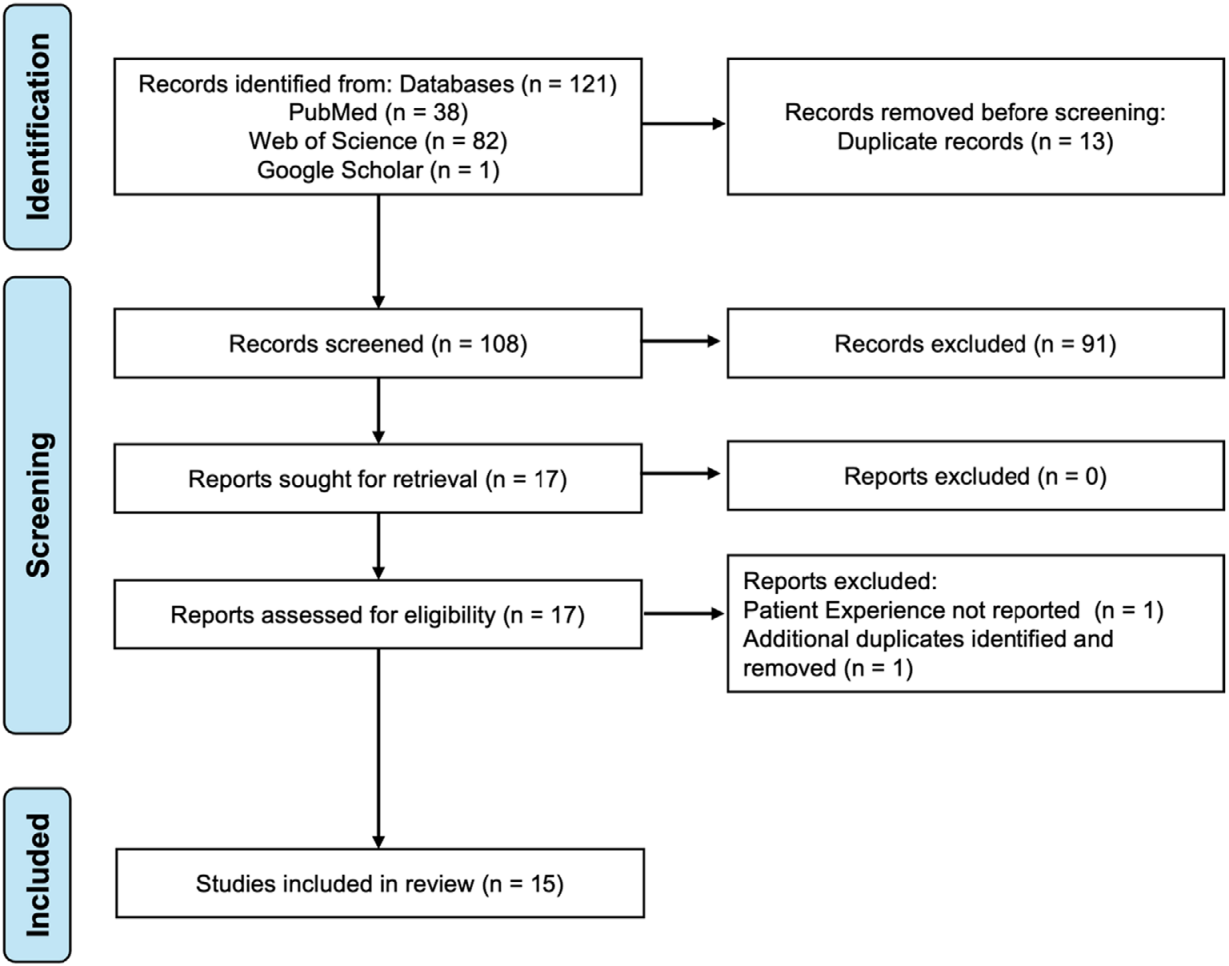
PRISMA flow chart diagram.

The literature search identified 121 records from PubMed (*n* = 38), Web of Science (*n* = 82), and Google Scholar (*n* = 1). After removing 13 duplicates, 108 records remained for further screening. During the primary screening, 91 records were excluded based on their title, abstract, and publication type. The secondary screening involved a full‐text review, resulting in the exclusion of two additional records. Ultimately, 15 studies^5^, ^18^, ^19^, ^20^, ^21^, ^22^, ^23^, ^24^, ^25^, ^26^, ^27^, ^28^, ^29^, ^30^, ^31^ were included in the review (Figure 1).

### 3.2 Characteristics of the included studies

A summary of the included studies is presented in Table 1. Of the 15 studies, one was a review article.^5^ No eligible dissertations, conference proceedings, or protocols were identified. The studies were published between 2008 and 2024, with research conducted in Australia (*n* = 4),^18^, ^20^, ^27^, ^30^ the United Kingdom (*n* = 3),^19^, ^21^, ^28^ the United States (*n* = 3),^5^, ^26^, ^29^ China (*n* = 1),^22^ Singapore (*n* = 1),^23^ Pakistan (*n* = 1),^24^ Belgium (*n* = 1),^25^ and the Netherlands (*n* = 1).^31^ Surveys were conducted in the official language of each country. Although most surveys were conducted in English, some were conducted in Chinese,^22^, ^23^ Urdu,^24^ Dutch,^25^, ^31^ and Welsh.^28^

**TABLE 1:** Characteristics of included articles (*n* = 15).

Author	Year	Country	Language	Participants	Research methods	PREM	Key findings
Pasquier et al.^18^	2008	Australia	English	Consumer NCDs	Qualitative Interviews (FG)		In primary care adherence support, pharmacists often provide insufficient information, leading many patients to expect more guidance from physicians
Loweir et al.^19^	2013	UK	English	Patient Cardiovascular diseases & Pharmacist	Qualitative Interviews (Individual)		The knowledge and understanding of pharmacists regarding the services they provide to patients with heart failure were deemed adequate, and the delivery of these services in community pharmacies was considered appropriate
Knox et al.^20^	2014	Australia	English	Consumer Mental illnesses & Carer	Qualitative Interviews (Individual)		Although patients appreciated suggestions for expanding the information provided by pharmacists and improving pharmacy environment, individualized and respectful care remained their highest priority
Kayyali et al.^21^	2018	UK	English	Patient Medication review	Mixed Questionnaire & Interviews (Individual)		Patients with long‐term conditions reported a lack of detailed medication counseling and minimal involvement in shared decision‐making (SDM) during medication review services at community pharmacies
Chen et al.^22^	2018	China	Chinese	Consumer OTC medicine purchasers	Qualitative Interviews (Individual)		Consumers purchasing over‐the‐counter (OTC) medications at community pharmacies expressed a desire for more appropriate advice from pharmacists regarding medication selection and usage. Some of these consumers voiced distrust in pharmacists' knowledge and communication skills
Siaw et al.^23^	2018	Singapore	Chinese	Patient Diabetes	Qualitative Interviews (FG)		Patients receiving diabetes care services managed by pharmacists at community pharmacies found explanations about medication usage, side effects, and management strategies to be helpful, as well as support in improving medication adherence
Asif et al.^24^	2019	Pakistan	Urdu	Customer	Qualitative Interviews (Individual)		Users of chain community pharmacies highly valued the counseling and medication labelling services provided, though they expressed concerns about alternatives and stock shortages
Wuyts et al.^25^	2020	Belgium	Dutch	Patient Older polymedicated patients	Quantitative Questionnaire	✔	A medication review conducted by pharmacists for elderly polypharmacy patients at home considerably reduced the medication burden. The Living with Medicines Questionnaire (LMQ) was reported as a promising scale for evaluating elderly polypharmacy patients
Carpenter et al.^26^	2021	USA	English	Patient NCDs	Mixed Questionnaire & Interviews (Individual)	✔	The developed patient experience evaluation scale effectively captured the relationship between the quality of pharmacy services and the duration of visits, demonstrating excellent reliability and validity
Carter et al.^27^	2021	Australia	English	Patient	Quantitative Questionnaire	✔	Patients who scored low on the patient experience scale (pSQS), indicating a perception of poor‐quality community pharmacy services, also reported lower levels of self‐reported medication adherence
Mantzourani Efi et al.^28^	2021	UK	English & Welsh	Patient Sore throat test and treat	Quantitative Questionnaire		Patients who received the sore throat test and treat (STTT) service from pharmacists highly rated access to the pharmacy and the pharmacists' expertise, suggesting a potential shift in health‐seeking behavior from General Practitioners to pharmacists
Murry et al.^29^	2021	USA	English	Patient Medicare part D	Quantitative Questionnaire		When selecting Medicare Part D services at the pharmacy, patients faced challenges in gathering information and assessing reliability, despite having a high level of awareness of the plans’ features and differences
Carter et al.^30^	2022	Australia	English	Patient	Quantitative Questionnaire	✔	The 19‐item pSQS, with one item excluded from the original version, can be effectively used in both field and online surveys. Moreover, the shortened version, pSQS‐SF6, retained the same measuring ability as the full pSQS
Murry et al.^5^	2023	USA	English	N/A	Review		Focusing on patient experience is crucial for designing and evaluating effective services in patient‐centered care
Davies et al.^31^	2024	Netherlands	Dutch	Patient Long‐term users of opioids	Qualitative Interviews (Individual)		Patients using opioids long‐term reported a lack of counseling on opioid risks, follow‐up evaluations for refill prescriptions, and communication regarding tapering

Abbreviations: FG, focus group; N/A, not applicable; NCDs, noncommunicable diseases; PREM, patient‐reported experience measures; UK, the United Kingdom; USA, the United States of America.

Regarding participant terminology, studies referred to them as “patients” (*n* = 10),^19^, ^21^, ^23^, ^25^, ^26^, ^27^, ^28^, ^29^, ^30^, ^31^ “consumers” (*n* = 3),^18^, ^20^, ^22^ and “customers” (*n* = 1).^24^ In addition, two studies included both patients and other stakeholders, such as pharmacists^19^ and caregivers.^20^

The methodologies employed in these studies included qualitative (*n* = 7),^18^, ^19^, ^20^, ^22^, ^23^, ^24^, ^31^ quantitative (*n* = 5),^25^, ^27^, ^28^, ^29^, ^30^ and mixed (*n* = 2).^21^, ^26^

### 3.3 Characteristics of PREMs

Four quantitative studies focused on PREMs^25^, ^26^, ^27^, ^30^ (Table 2). Two of these developed new PREMs,^26^, ^30^ whereas the remaining two utilized existing scales.^25^, ^26^, ^27^ The number of items in the scales ranged from six to 41, with response options based on 5‐ or 7‐point Likert scales.

**TABLE 2:** Characteristics of PREMs (*n* = 4).

Name of PREM	Year	Country	Measurement	Content	Items	Response
Living with Medicines Questionnaire (LMQ) version 3.0^24^	2020	Belgium	Burden of long‐term medicine use	1. Patient**–**healthcare professional relationships and communication about medicines 2. Practical difficulties 3. Cost‐related burden 4. Side effects burden 5. Effectiveness 6. Concerns about medicine use 7. Interferenc**e** with daily life 8. Autonomy/control over medicines	41	Likert scale ‐ 5‐point
The 7‐item patient experience measure^25^	2021	USA	Quality of community pharmacy services^a^; review, dispense, and administer	The 7‐item patient experience measure assessed the experiences of patients with their pharmacies, pharmacists, and the support and quality of medication‐related services that their pharmacists provided.	7	Likert scale ‐ 5‐point
The Perceived Service Quality Scale (pSQS)^26^, ^29^	2021 2022	Australia	Quality of community pharmacy services^a^; review, dispense, administer, and pharmacy environment	1. Health and Medicines Advice 2. Nonprescription Service 3. Relationship Quality 4. Technical Quality 5. Environmental Quality 6. Health Outcome	19	Likert scale ‐ 7‐point
The short‐form of pSQS (pSQS‐SF6)^29^	2022	Australia	Quality of community pharmacy services^a^; review, dispense, administer, and pharmacy environment	1. Health and Medicines Advice 2. Nonprescription Service 3. Relationship Quality 4. Technical Quality 5. Environmental Quality 6. Health Outcome	6	Likert scale ‐ 7‐point

Abbreviations: PREM, patient‐reported experience measures; USA, United States of America.

^a^ Community pharmacy services; review, prescribe, dispense, and administer.^16^

The Living with Medicines Questionnaire, used in one study,^25^ was a PREM designed to assess the burden of pharmacotherapy and was translated into Dutch.^32^ The other PREMs focused on patient experiences with common pharmacy services.^26^, ^27^, ^30^ Notably, the Perceived Service Quality Scale (pSQS), developed in Australia, has been implemented in pharmacy practice. In addition, a shortened version, the pSQS Short‐Form Version (pSQS‐SF6), was developed,^30^ along with investigations into its relationship with medication adherence.^27^

## 4 DISCUSSION

### 4.1 Summary of evidence

This scoping review examined patient experience in community pharmacies by analyzing 15 studies. Among the included studies, definitions of “patient experience” and “patient satisfaction” were often conflated, although a shift toward the former was observed over time. Even studies explicitly evaluating patient experience often relied on subjective measures, such as patient satisfaction and expectations.^20^, ^22^, ^24^, ^28^, ^29^ This trend aligns with findings from broader studies on patient experience and satisfaction across various healthcare settings.^12^, ^33^ However, the recognition of patient experience as a distinct concept only began to emerge in the late 2000s, gaining increasing attention in recent years. Unlike patient satisfaction, which often reflects expectations and personal preferences, patient experience offers a more objective assessment of care quality.^34^ As awareness of its importance grows, its broader application in pharmacy settings is expected to expand further.

The reviewed studies employed both qualitative and quantitative methodologies, although studies specifically addressing PREMs were limited. This review identified four studies on PREMs conducted in Australia,^27^, ^30^ the United States,^26^ and Belgium,^25^ predominantly from English‐speaking countries. Patient experience assesses the process of receiving healthcare services and their quality. As a quantitative tool, PREMs are particularly valuable for identifying specific areas for improvement and guiding service enhancements.

Community pharmacy services differ across countries owing to variations in national regulations, healthcare systems, and societal needs. Despite these differences, core functions—such as medication review, prescribing, dispensing, and administration—are consistently recognized as fundamental roles of community pharmacists worldwide.^16^ This review focused on patient experience in relation to these universally acknowledged aspects of pharmacy practice. Evaluating patient experience through this common framework enables cross‐national comparisons and contributes to the advancement of global pharmacy research. However, how patients perceive and report their experiences can be shaped by linguistic, cultural, and contextual factors. Thus, to ensure meaningful and accurate assessments, PREMs must not only be valid and reliable but must also be sensitive and adaptable to the cultural and linguistic contexts in which they are applied.^13^, ^35^, ^36^

The use of standardized measurement tools facilitates objective data analysis, enabling service improvements and comparative evaluations across different pharmacy settings. Of the four identified PREMs, studies from Australia and the United States focused on evaluating patient experience throughout the pharmacy visit process. In particular, the pSQS, developed in Australia, has undergone reliability and validity testing, and its short‐form version, the pSQS‐SF6, was subsequently developed.^30^ Furthermore, the pSQS has been applied in studies investigating its relationship with medication adherence.^27^ Moving forward, refining and adapting standardized scales such as the pSQS is essential to accurately capture patient experiences and generate actionable insights for service improvement.^37^ By adopting a person‐centered approach, community pharmacies can enhance patient engagement, contribute to improved health outcomes, and support the development of a more person‐centered healthcare system.

### 4.2 Limitations

This scoping review is the first comprehensive analysis of studies on patient experience in community pharmacies, highlighting existing gaps in the literature. The review adhered to established reporting guidelines, utilized major academic databases, and applied clear selection criteria. However, this study had some limitations. First, the research strategy was constrained by predefined keywords and selection criteria, which may have led to the exclusion of relevant studies. Second, patient experience studies published as facility or organizational reports instead of research articles may not have been identified. Finally, this review was restricted to studies published in English or Japanese. Articles published in other languages may have been overlooked, limiting the comprehensiveness of the findings. Despite these limitations, this review provides a foundation for future studies, emphasizing the need for standardized PREMs and further investigation of patient experience across diverse healthcare systems.

## 5 CONCLUSIONS

Although studies on patient experience are expanding, the use of PREMs remains limited. To enhance pharmacy services, standardized and culturally adaptable PREMs should be developed and implemented. This will strengthen person‐centered care, drive service improvements, and potentially contribute to better health outcomes and more sustainable healthcare systems.

## AUTHOR CONTRIBUTIONS

**Chiho Kaneko:** Conceptualization; data curation; formal analysis; investigation; methodology; project administration; resources; visualization; writing – original draft; writing – review and editing. **Shota Suzuki:** Conceptualization; funding acquisition; investigation; methodology; project administration; validation; writing – original draft; writing – review and editing. **Ibuki Tanaka:** Investigation; data curation; formal analysis; writing – review and editing; resources. **Haruna Hiruma:** Investigation; writing – review and editing; formal analysis; data curation; resources. **Hiroshi Okada:** Conceptualization; methodology; project administration; supervision; validation; writing – review and editing.

## FUNDING INFORMATION

This work was supported by the Pfizer Health Research Foundation [grant numbers 23‐9‐024].

## CONFLICT OF INTEREST STATEMENT

CK, SS, IT, and HH declare they have no conflicts of interest. HO has received donations from Yuyama Co., Ltd., Ishizuka Co., Ltd., Frant Co., Ltd., Neoplus Pharma Co., Ltd., and Solamichi System Inc.

## ETHICS STATEMENT

Ethics approval statement: None.

Patient consent statement: None.

Clinical trial registration: None.

## Data Availability

The datasets generated during and/or analyzed during the study are available from the corresponding author upon reasonable request.

## APPENDIX A

**TABLE A1:** Databases searched and search strategy (Search date: October 16, 2024).

Database	Combination of terms
PubMed	("patient‐centered"[Title/Abstract] OR "patient‐reported"[Title/Abstract] OR "client‐centered"[Title/Abstract] OR "client‐reported"[Title/Abstract] OR "consumer‐centered"[Title/Abstract] OR "consumer‐reported"[Title/Abstract] OR "customer‐centered"[Title/Abstract] OR "customer‐reported"[Title/Abstract]) AND "experience"[Title/Abstract] AND "community pharmac*"[Title/Abstract] AND ("service*"[Title/Abstract] OR "program*"[Title/Abstract] OR "intervention*"[Title/Abstract] OR "care*"[Title/Abstract] OR "consultation*"[Title/Abstract]) AND ("investigat*"[Title/Abstract] OR "survey*"[Title/Abstract] OR "reserach*"[Title/Abstract] OR "evaluat*"[Title/Abstract] OR "assess*"[Title/Abstract] OR "measur*"[Title/Abstract] OR "trial*"[Title/Abstract] OR "stud*"[Title/Abstract] OR "analy*"[Title/Abstract])
Web of Science	(patient‐centered (Topic) or patient‐reported (Topic) or client‐centered (Topic) or client‐reported (Topic) or consumer‐centered (Topic) or consumer‐reported (Topic) or customer‐centered (Topic) or customer‐reported (Topic)) AND (experience (Topic)) AND ("community pharmac*" (Topic)) AND (service* (Topic) or program* (Topic) or intervention* (Topic) or care* (Topic) or consultation* (Topic)) AND (investigat* (Topic) or survey* (Topic) or research* (Topic) or evaluat* (Topic) or assess* (Topic) or measur* (Topic) or trial* (Topic) or stud* (Topic) or analy* (Topic))
Google Scholar	allintitle:(patient‐centered OR patient‐reported OR client‐centered OR client‐reported OR consumer‐centered OR consumer‐reported OR customer‐centered OR customer‐reported) experience ("community pharmacy" OR "community pharmacist")
